# The current stage of Italy in the implementation of genomics into the National Healthcare System: an application of the B1MG maturity level model

**DOI:** 10.3389/fpubh.2025.1425990

**Published:** 2025-04-16

**Authors:** Valentina Baccolini, Erica Pitini, Daniela Galeone, Carolina Marzuillo, Americo Cicchetti, Marcello Arca, Astrid M. Vicente, Stefania Boccia, Paolo Villari

**Affiliations:** ^1^Department of Public Health and Infectious Diseases, Sapienza University of Rome, Rome, Italy; ^2^Department of Translational and Precision Medicine, Sapienza University of Rome, Rome, Italy; ^3^General Directorate for Health Prevention, Ministry of Health, Rome, Italy; ^4^Alta Scuola di Economia e Management dei Sistemi Sanitari (ALTEMS), Università Cattolica del Sacro Cuore, Rome, Italy; ^5^Departamento de Promoção da Saúde e Prevenção de Doenças Não-Transmissíveis, Instituto Nacional de Saúde Doutor Ricardo Jorge, Lisboa, Portugal; ^6^Section of Hygiene, Department of Life Sciences and Public Health, Università Cattolica del Sacro Cuore, Rome, Italy; ^7^Department of Woman and Child Health and Public Health, Fondazione Policlinico Universitario A. Gemelli IRCCS, Rome, Italy

**Keywords:** genomics, implementation, national healthcare system, expert consultation, Italy

## Abstract

**Introduction:**

Genomics holds significant promise for prevention and clinical care yet integrating it into the national healthcare system (NHS) requires considerable system-wide changes. This study assessed the current stage of Italy in the use of genomics, to map critical areas for improvement and contribute to a strategic plan.

**Methods:**

A total of 18 experts rated individually the level of maturity of the Italian NHS on a scale from 1 (lowest) to 5 (highest) using the B1MG Maturity Level Model tool. This instrument is an European matrix of 49 indicators grouped into eight domains: governance, economic aspects, ethics and legislation, public awareness, workforce skills, clinical organization, clinical guidelines, and data infrastructure. Consensus procedures were performed within each domain to finally agree on one maturity level per indicator.

**Results:**

Despite a few national initiatives, Italy shows a local level of implementation in most indicators. Genomic medicine is considered a priority, but still lacks an updated strategy and investment plans. A higher maturity is reached for ethical and legal aspects, but there is a strong need to invest in workforce training, citizen engagement and literacy, and large-scale adoption of tools and novel technologies. Infrastructures and guidelines to improve data storage, management, analysis, interpretation, and sharing are not yet widespread available.

**Discussion:**

Italy is at the beginning of its journey towards a sustainable implementation of genomics. An updated national strategy with coordinated actions and investment plans is needed to make progress in key areas, including personnel education, public engagement, technical infrastructure, and clinical organization.

## Introduction

The potential for genomics, which involves using a person’s genomic information to guide their clinical care, has long been acknowledged as a transformative approach in modern medicine (1). Rapid advances in laboratory technologies allow to acquire a large set of genomic data that can be used to optimize the healthcare decision-making process (2). Accordingly, many scenarios for clinical use of information about a patient’s genome have been proposed (3), including prognostic, predictive, diagnostic, screening and pharmacogenomic testing, which can span across multiple life stages (4) and many specialties of medicine (5). However, despite this increasing relevance to healthcare and their decreasing cost (6), translating the results of genomic research into clinical practice is still slow (3) and often limited to single institutions and/or applications (7), leading to implementation disparities and differences in access to care (8).

This is because widespread integration of genomic medicine in healthcare requires system-wide change (9, 10). Considerable investments are needed in key areas, including the technical infrastructure, personnel, and organization of healthcare delivery (9). The existing barriers have a different impact depending on the country (11, 12), but they refer to the same issues, including data integration and interpretation, workforce capacity and capability, evidence on effectiveness and cost-effectiveness, public acceptability, and ethical and legislative issues (12). For this reason, a few large scale sequencing projects have been started to better understand the clinical significance of genetic variations (13), global collaborations have been promoted to improve genomic data sharing (3, 14), and a few countries, such as Australia (15), France (16), or the United Kingdom (17), have designed frameworks to support their national healthcare system (NHS) in the introduction of genomic applications. For instance, Australia recently developed the National Health Genomics Policy Framework, which was successively endorsed by the Council of Australian Governments Health Council. This framework delivers a strong and coherent structure for coordinating activities across jurisdictions (15). Similarly, France and the United Kingdom conceived national plans for genomic medicine to set out priority actions, organize new pathways of care and counseling, make decisions about insurance coverage, and increase financial investments (16, 17).

Despite all these efforts, the implementation process is still demanding (12), and every country faces its own healthcare context (7, 18). Italy is no exception: it was a pioneer in the development of public health genomic policies, and in 2013 and 2017, it implemented two national plans and developed a national pathway for the evaluation of genomic applications (19). Nevertheless, it struggles with the adoption of these technologies like other countries (19, 20), a challenge further complicated by Italy’s decentralized healthcare system, where the Regions are responsible for organizing and delivering health services (21–24). Within this context, the identification of barriers is crucial to effectively integrating genomics in clinical practice (11), especially within the recent normative framework aimed at transforming primary care into community care, overcoming geographical disparities, and achieving greater effectiveness of services (25). Therefore, since a comprehensive analysis of the current stage of genomic implementation in Italy was still lacking, we applied the Beyond 1 Million Genome (B1MG) Maturity Level Model (MLM) tool (26) to assess the maturity of its NHS in the use of genomics. The aim was to map critical areas for improvement and ultimately contribute to the development of an action plan for progress toward sustainable optimization.

## Materials and methods

### The MLM tool

The MLM framework was elaborated in early 2022 by the European B1MG initiative as a support tool for countries to self-assess their maturity in the genomic integration process. The development process is described elsewhere (26). Briefly, after a literature review of relevant papers on maturity level frameworks developed in the field of healthcare and a few experts’ consultations, the final tool was defined after a two-round Delphi process.

The tool is public available matrix^1^ (26) that consists of 49 indicators grouped into 41 subdomains. These subdomains are then categorized into eight domains, each with the aim of evaluating the operational and organizational levels reached at national (or subnational, if applicable to the country) areas deemed relevant for a successful implementation on genomics into the healthcare system: (1) governance and strategy; (2) investment and economic model; (3) ethics, legislation, and policy; (4) public awareness and acceptance; (5) workforce skills and organization; (6) clinical organization, infrastructure, and tools; (7) clinical genomics guidelines and infrastructure; and (8) data management, standards, and infrastructure.

For each indicator, a group of selected experts must rate the level of maturity of their country on a scale from 1 (lowest) to 5 (highest). Each indicator has its own levels, but in general terms, the levels are:

- Level 1: *ad hoc* or no implementation
- Level 2: defined at local level
- Level 3: documented, functional, and monitored
- Level 4: adopted by national healthcare system
- Level 5: adaptable to opportunity and change and supporting international cooperation.

Each expert, as a stakeholder representative, must individually respond to one or more domains, according to expertise and scope of activities, provide the rationale for the self-assessment, and list the evidence to refer to that supports the maturity level selection. Once the experts have completed their assessments, results and evidence must be collectively discussed to reach a consensus on the maturity level per indicator. The decision rules and procedures in consensus meetings are left to the country to decide (26).

### The assessment process and data analysis

**Figure 1:**
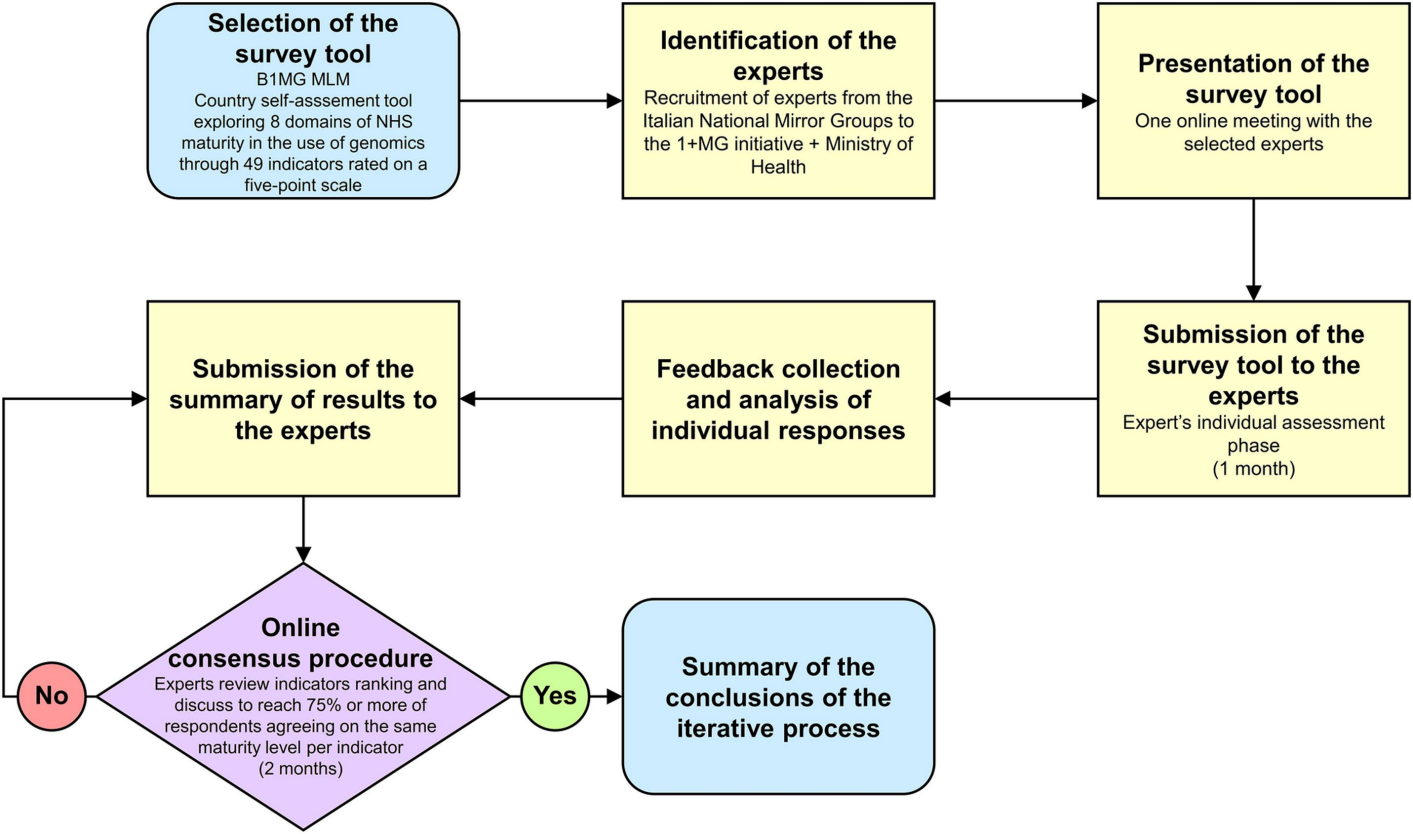
Workflow of the assessment process. MLM, Maturity Level Model. 1 + MG, 1 + Million Genomes. B1MG, Beyond 1 + MG.

To identify the experts responsible for the assessments we used a network created by a national project entitled “Italian Genomics Strategy” (27). Such a project, funded by the Ministry of Health, aimed to financially support the institutions and the professionals that are involved in the B1MG initiative to ultimately contribute to the development of a national genomics strategy. This network was chosen for the purposes of this study because it gathered together 16 institutions, including the leaders of the National Mirror Groups (NMGs) involved in the B1MG initiative, therefore the most prominent Italian scientific and technical experts in genomics (Figure 1).

During one of the project’s monthly meetings, the MLM tool was officially presented to all partners. Participants were then asked to declare the domain(s) they were willing to assess in a survey that took place 1 week after. Then, a MLM project kick-off meeting was scheduled to discuss deadlines, tasks, and procedures and a few days later, experts were provided via e-mail the assessment tool with the detailed instructions on the evaluation process. They had 1 month to conduct the individual assessment phase, but reminders were sent on weekly basis. After the collection of all individual assessments, inputs were qualitatively combined together, and each expert received by email an anonymized summary of the domain results he/she had assessed as stakeholder (i.e., all assessments received per indicator). Lastly, online consensus procedures were carried out within each domain over a two-month period. During these meetings, each expert had to firstly motivate his/her maturity level selection, examining the evidence and the rationale on which had been based the evaluation, and secondly, if necessary, open debates between different evaluations were started, with the aim of finally agreeing on a maturity level per indicator. Consensus was defined as 75% or more of respondents agreeing on the same maturity level.

The Ethics Committee for Transdisciplinary Research of Sapienza University of Rome approved this study (ID: 97/2023).

## Results

The assessment team consisted of 18 experts. They were mostly males (66.7%) and from academia (72.2%). Participants were working in institutions located in either central (61.1%) or northern Italy (38.9%). Each domain was assessed by 4.8 experts on average (min/max: 2/10). Complete consensus (i.e., 100% agreed on the final level of maturity) was reached in all indicators after a mean of 1.3 meetings per domain (min/max: 1/2).

### Domain 1—Governance and strategy

**Figure 2:**
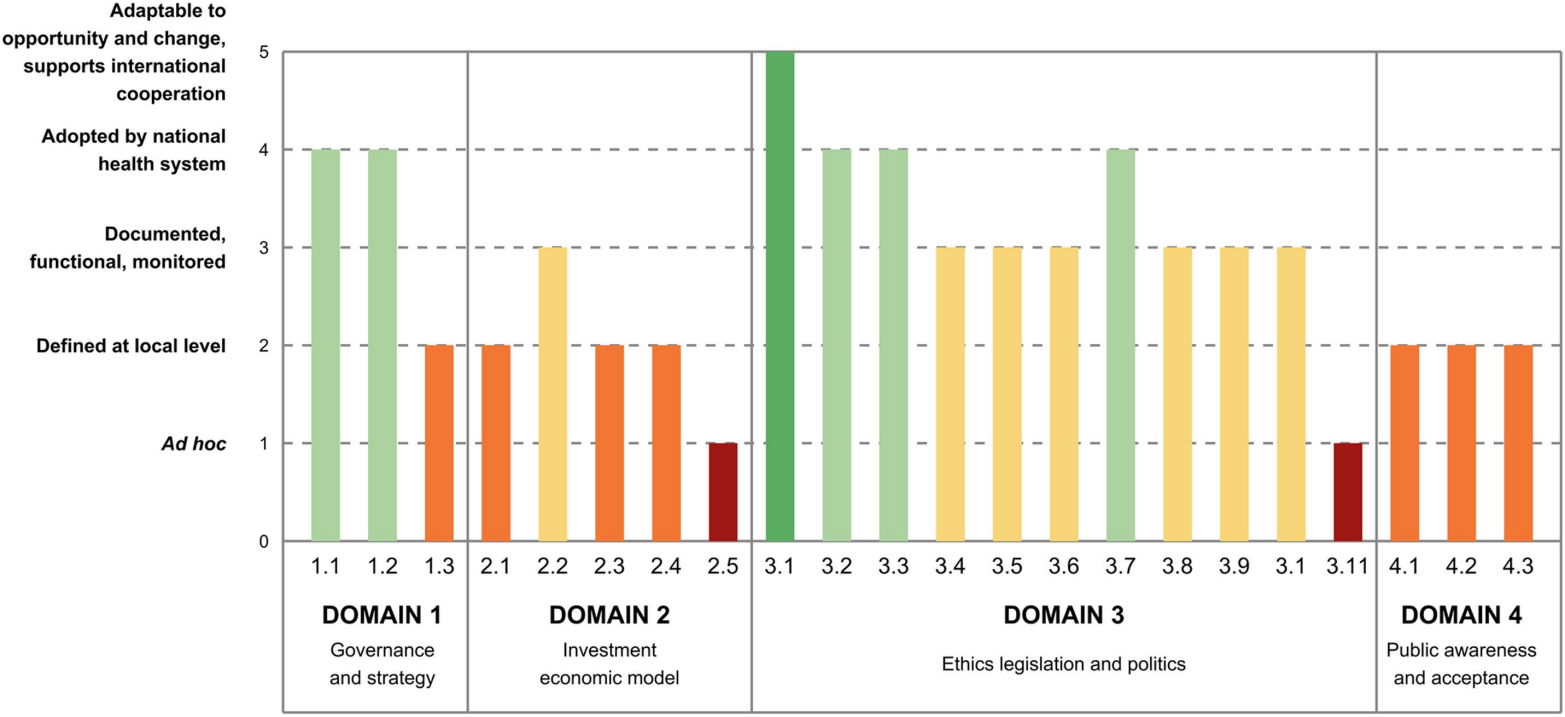
Italy’s maturity level in the implementation of genomics by indicator (Domain 1 to 4). 1.1. Governance—country has a dedicated governance for genomics in healthcare. 1.2. Priority—genomics in healthcare is established as a priority at national level. 1.3. Strategy—there is a national strategy for genomics in healthcare with a costed implementation plan. 2.1. Investment—there is an investment plan at the national level for genomics in healthcare, with public or mixed public-private funding models. 2.2. Access and reimbursement—there is a framework for reimbursement or no-cost access plans for genomic tests, at the national level. 2.3. Health Economics—there is a HTA framework to assess genomic tests in healthcare. 2.4. Health Economics—there is a framework for cost-effectiveness assessment of genomic tests. 2.5. Health Economics—societal benefits are considered in economic modeling for genomic medicine. 3.1. Data protection and privacy—there are norms to protect and ensure the lawful, fair, and transparent processing of personal data. 3.2. Data protection and privacy—there are norms protecting the confidentiality of patient genetic/genomic test results, and specifically clarifying where family members may have rights to access these results. 3.3. Data protection and privacy—there are norms limiting genetic/genomic testing to legitimate purposes and preventing misuse (e.g., no employer/insurer discrimination). 3.4. Consent to genetic/genomic testing—there are norms to ensure appropriate consent is obtained and counseling is provided in relation to genetic/genomic testing. 3.5. Consent to genetic/genomic testing—there are special rules to ensure that vulnerable groups have access to genetic/genomic testing, with counseling and appropriate protections to fully respect their rights and avoid their exploitation. 3.6. Quality of patient care involving genetic/genomic testing—there are norms ensuring the quality of genetic/genomic testing services (e.g., professional codes, self-regulatory bodies). 3.7. Health data sharing and reuse—there are norms addressing the accreditation, registration, supervision, secure storage, and responsible use (including exchange and sharing) of human biological samples. 3.8. Health data sharing and reuse—there is a national strategy for promoting health research and innovation, and associated data protection rules allowing sharing and further processing of health/genetic data for research or treating other patients. 3.9. Health data sharing and reuse—there are norms facilitating genomic data sharing by researchers and/or healthcare providers, at the national and international levels. 3.10. Research ethics—there are norms and processes ensuring the ethical practice and scientific integrity of genomic research. 3.11. Research ethics—there is a national research ethics committee or network to effectively and efficiently oversee the conduct of multicenter genetic/genomic studies. 4.1. Awareness—there are literacy programs or campaigns on genomic medicine with monitored impact on awareness. 4.2. Acceptance—synergies with patient associations are well established. 4.3. Communication to the general public—there is a communication strategy for genomic medicine.

This implementation level was judged quite high for the governance and priority subdomains (Figure 2). Indeed, two national plans were issued between 2013 and 2017, which laid the foundation for the governance of genomics in healthcare, defining priority actions and actors, although not all actions have been fully implemented to date and institutional actors are not yet completely operational (19) (1.1). Similarly, experts stated that genomics is considered as a priority in Italy, as it is included also in other relevant national strategies, such as the last National Prevention Plans (19), even though an incomplete and heterogeneous implementation across the Regions has emerged (1.2). By contrast, a lower level of maturity was found in the last subdomain, in which a national strategy for genomics with a costed implementation plan is still in the early stage (1.3).

### Domain 2—Investment and economic model

Relatively low levels of implementation were found in this domain: investment plans are still under development (2.1), whereas the reimbursement of healthcare pathways and models are in place for some genomic conditions only (2.2) (Figure 2). As for the health economics subdomain, Health Technology Assessment (HTA) and cost-effectiveness frameworks to assess genomic tests in healthcare are under development (2.3, 2.4): Italy elaborated a methodological framework for assessing genomic tests, but it has yet to be validated (28). Efforts are now directed on creating a comprehensive HTA framework for genomic testing, including prioritization, evaluation, and appraisal phases and defining an institutional network for genomic tests assessment (19, 29). Likewise, despite many European initiatives in which Italy is participating, a cost-effectiveness model is still being developed. Lastly, societal benefits were considered at an early level of maturity in the economic modeling since the framework is still under development (2.5).

### Domain 3—Ethics, legislation, and policy

The regulatory framework in Italy is formed by the rules directly applicable of the European General Data Protection Regulation and the legislative decree n.196/2003 for the residual indications, that together ensure that norms to protect the lawful, fair, and transparent processing of personal data are implemented, enforced, and fit-for-purpose (3.1) (Figure 2). As for the confidentiality of patient test results, the norms were deemed implemented and consistently enforced (3.2). Similarly, norms restricting genomic testing to valid purposes and avoiding misuse were judged effectively implemented (3.3). With regard to consent to genomic testing, the experts proposed an intermediate level of maturity, being rules implemented but not consistently applied (3.4). Specifically, while in Italy there is no specific law governing the prescription of genetic tests and related counseling, guidelines have been issued over the years concerning the diagnosis and prevention of genetic diseases, genetic counseling, and the protection of individuals undergoing genetic testing. Besides, some specific regulations exist for minors, and tailored guidelines ensure vulnerable subjects receive adequate protection to safeguard their rights and prevent exploitation (3.5), even though they are not consistently enforced. The maturity level assigned to norms ensuring the quality of genomic testing services was deemed intermediate (3.6), as there is no specific law, but guidelines have been issued over the years supplemented by *ad hoc* opinions focusing on improving quality, adopting a multidisciplinary approach, and monitoring the use of genetic tests. Norms governing the sharing and reuse of health data, particularly concerning human biological samples, were reported to be implemented and consistently enforced (3.7). Indeed, Italian regulations cover the collection, registration, secure storage, and responsible use of such samples. Biobanks, responsible for managing human biological materials, adhere to quality and safety standards for donation, procurement, processing, storage, and distribution. The indicators concerning norms on national strategies for promoting health research allowing sharing and further processing of health data (3.8) and genomic data sharing (3.9) show that while they are established, their enforcement is limited and mostly related to research projects. Similarly, norms ensuring the ethical practice and scientific integrity of genomic research were deemed implemented but not fully enforced (3.10). Indeed, despite existing guidelines regarding the ethical practice and scientific integrity of research, in Italy there is no specific guideline regarding the ethics of genomic research, even though some opinions of the Italian Committee for Bioethics and the Italian Committee for Biosafety, Biotechnologies and Life Sciences have been expressed over the years. Lastly, although these bodies support the government in the elaboration of scientific, social security and consultancy guidelines at the national level, a national committee or a network that oversees the conduction of multicenter genomic studies does not exist yet (3.11).

### Domain 4—Public awareness and acceptance

Experts indicated that all aspects related to public awareness, acceptance, and communication stand at a local level of implementation (4.1, 4.2, 4.3; Figure 2). Indeed, despite a few national projects (30, 31) or initiatives (32) laying the groundwork for literacy and communication campaigns or establishing connections with patient associations, there are only bottom-up initiatives.

### Domain 5—Workforce skills and organization

**Figure 3:**
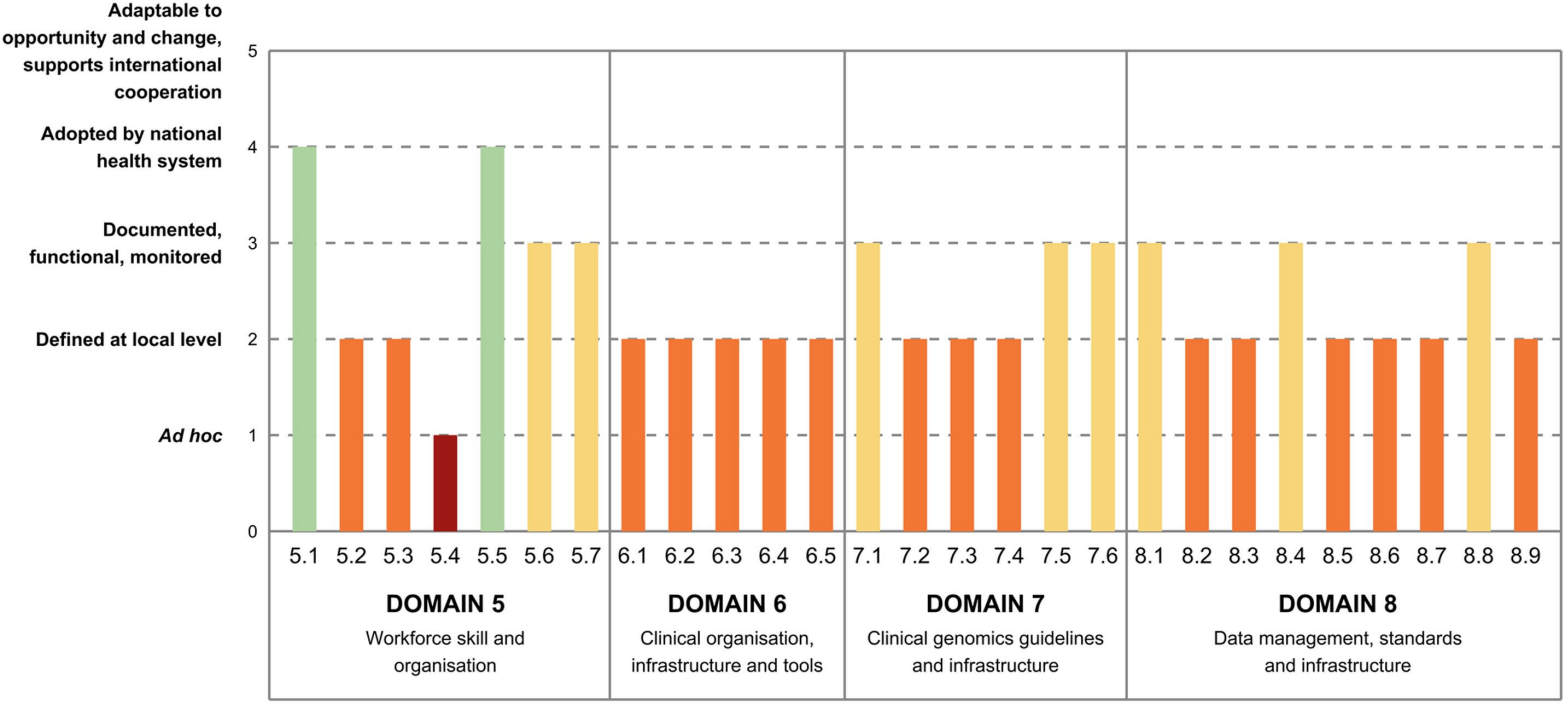
Italy’s maturity level in the implementation of genomics by indicator (Domain 5 to 8). 5.1. Education—genomics is integrated in general university curricula for medical doctors. 5.2. Education—genomics is integrated in general curricula for nurses. 5.3. Education—genomics is integrated in general curricula for pharmacists. 5.4. Careers in genomic medicine—there are officially recognized professional titles and career paths for genomic medicine. 5.5. Careers in genomic medicine—there are training programs for genetic counseling. 5.6. Careers in genomic medicine—there are life-long or continuing education programs in genomic medicine for different healthcare professionals. 5.7. Policy makers—there are programmes for policy makers and healthcare managers to raise awareness on genomic medicine and its implications for healthcare. 6.1. Information and Communications Technology (ICT) tools for clinical decision—there are ICT tools supporting clinical interpretation of genomic results, clinical decision-making and communication with the patient implemented in public hospitals and clinics. 6.2. Multidisciplinary teams—clinical teams for genomic medicine are multidisciplinary and include ICT, biomedical and psychology experts. 6.3. Uptake of novel tools and technologies for genomics—adoption of novel technologies and software tools to support clinical decisions is fit for purpose. 6.4. Synergies with research—there are processes established for the integration of the clinics with research outcomes. 6.5. Partnership with industry—there are effective partnerships with stakeholders from the industry sector. 7.1. Sequencing/genotyping infrastructure—genomic centers are established. 7.2. Sequencing guidelines—guidelines for sequencing are defined. 7.3. Primary bioinformatics analysis—guidelines for genomic data analysis are defined. 7.4. Structure of sequence-associated metadata—guidelines for sequence-associated metadata structure to support clinical interpretation are established. 7.5. Clinical interpretation—guidelines for clinical interpretation of genomic results are defined. 7.6. Clinical reporting—guidelines for clinical reporting of genomic results are defined. 8.1. Data security—infrastructure and policies for data security are established. 8.2. Data discoverability (findable)—guidelines for structuring metadata for datasets are established. 8.3. Data access management (accessible)—data access governance framework is established. 8.4. Data access management (accessible)—data sharing policies and data flows are established. 8.5. Reception and interfaces (interoperable)—guidelines for record level data structure are established. 8.6. Reception and interfaces (interoperable)—guidelines for dataset structure are established. 8.7. Reception and interfaces (interoperable)—data sharing infrastructure is established using a federated model. 8.8. Reception and interfaces (interoperable)—services for data reception to support interoperability are established. 8.9. Processing and analysis (reusable)—computational and data infrastructure for medical reuse and secondary data analysis is available.

While genomic training for medical doctors, encompassing genetics, is widely integrated into university curricula (5.1), similar provisions for nurses and pharmacists are still in the development stage, with needs assessment and gap identification being underway (5.2, 5.3; Figure 3). There is currently no defined workforce pathway specifically recognizing genomic medicine professionals in general curricula, but it exists for geneticists (5.4). By contrast, training programs for genetic counseling are being implemented (5.5), along with continuing education initiatives for different healthcare professionals, even though they are under implementation (5.6). Similarly, targeted awareness-raising programs on genomic medicine are available for policymakers and healthcare managers, but their implementation is not uniform (5.7).

### Domain 6—Clinical organization, infrastructure, and tools

Experts have assessed all indicators in this area to have a relatively low level of maturity (Figure 3). Specifically, information and communication technology tools (6.1), multidisciplinary teams (6.2), and the utilization of innovative technologies for clinical decision support (6.3) are implemented in selected facilities only, often related to the participation in research projects, and not widely accessible. A few efforts to integrate clinics with research (6.4) and establish partnerships with the industry sector (6.5) are underway, albeit primarily conducted at local level.

### Domain 7—Clinical genomics guidelines and infrastructure

According to experts, infrastructure networks for clinical genomics are in the development stages in some Italian regions, with the establishment of common working guidelines and shared policies, but there is still a lack of national coordination (7.1; Figure 3). Guidelines for sequencing (7.2), genomic data analysis (7.3), and metadata structure (7.4) are defined and available only locally, but they draw upon best practice guidelines from international scientific societies. As for guidelines for clinical interpretation and reporting, they usually follow national and international societies’ best practices, but some disease areas are more advanced than others (7.5). Similarly, clinical reporting adheres to guidelines based on national and international best practices, although enforcement remains heterogeneous and depends on the local context (7.6).

### Domain 8—Data management, standards, and infrastructure

Data security (8.1) is ensured through nationally defined policies and infrastructures, but their enforcement remains inadequate (Figure 3). Guidelines for structuring and handling datasets are established at the local level only (8.2), similarly to policies governing data access (8.3), with stakeholder consultation occurring in selected institutions only. Sharing policies are standardized at the local level, but electronic management of data flows is limited (8.4). Regarding data reception and interfaces, guidelines for both record-level data structure (8.5) and dataset structure and access for discovery (8.6) are defined locally and are scarcely implemented across institutions, with each department or institution that typically establishes its own data sharing infrastructure (8.7). However, quality control measures, although it follows local indications, generally align with international standards (8.8). Lastly, in terms of data processing and analysis, computational and data infrastructure for medical reuse and secondary data analysis varies locally, with only a few regions having established networks (8.9).

## Discussion

Italy, like many other countries (3, 18), is navigating the complex interplay between rapidly evolving genomic technologies and the delivery of healthcare services within a publicly funded system. In line with these challenges, this study provided an overall picture in which the country stands at the beginning of its journey toward a sustainable implementation of genomics, with a few national initiatives that sought to coordinate activities and establish connections across institutions. One of the most advanced domains was that on regulations and norms, that are issued at national level to comply with the European laws and provide the legal framework for any collection, storage, and analysis of genomic data. A crucial aspect is represented by the informed consent, the legal basis on which to process personal data, and whose model (i.e., from quite restricted to broad authorization) affects the opportunity for health data sharing and reuse (33), two closely related issues around which there is a long-lasting international debate (34). Within this context, given the numerous benefits of collaboration between different institutions and jurisdictions in relation to pooling resources, expertise, and data (35), the lack of an ethical committee responsible for the conduction of multicenter studies has emerged as an important weakness that requires immediate consideration.

As for the government commitment, the importance of integrating genomics in the Italian NHS to improve patient outcomes has long been recognized, with the first national policy published in 2013 and a second plan approved a few years later (19, 36) that contributed to draft a governance for *omics* sciences and recommendations for the integration of genomics into prevention, diagnosis and care. Unfortunately, this plan was not complemented by a comprehensive funding scheme, but the fulfillment of some goals has been supported by the Ministry of Health through specific projects financed by the National Center for Disease Prevention and Control (27, 30). However, in addition to the lack of explicit financial planning, the integration of genomics into clinical and public health practice was further complicated by the paucity of evidence on the clinical utility of these technologies. Indeed, to address the methodological challenges in the assessment of their benefits and costs, that are not only those strictly related to health or the individual undergoing genetic testing (29, 37), several initiatives were funded at the national and international level, first of all the HEcoPerMed project (38), that studies economic models and reimbursement schemes for innovative treatments in personalized medicine, but these issues are far from being solved (19, 29). Nevertheless, the country is getting clear about its priorities, and several efforts are in place, including the present study, to start the definition of an updated genomic strategy, that should become available in the near future (27).

Two other evaluation domains regarded the main actors in healthcare delivery, patients and healthcare workers, whose lack of appreciation of the potential benefits of genomics slow the integration process (1). While there are countries, such as the United Kingdom or Finland (39), that have created educational programs to increase the competencies of all healthcare professionals, Italy has focused its efforts on physicians training. Therefore, it is necessary to strengthen the Italian healthcare system by improving the integrated health and social care management of patients through well-trained multi-professional teams, including all healthcare professionals involved in the genomic pathway (40). Similarly, unlike the United Kingdom or Estonia (39), that since the beginning have involved patients and citizens in relevant genomic decisions or have been conducting communication campaigns for years, Italy has paid limited attention to national awareness and information campaigns (30, 31). Given that both healthcare professionals and citizens play a key role in shaping the future of genomics (18), further efforts should be made to promote their engagement and participation in policymaking. Some investments should also be directed toward a large-scale adoption of software tools and novel technologies, that could help both clinicians and patients in their decision-making process (41). Likewise, the establishment of multidisciplinary teams, in addition to partnerships with research or the industry sector, can integrate expertise and resources, hereby facilitating comprehensive approaches to genomic research, interpretation, and application (42).

Notably, the need of enabling access to genomic data through a technical infrastructure that comply with the FAIR (i.e., findability, accessibility, interoperability, and reusability) Data Principles is a major milestone in the roadmap toward sustainable implementation. In Italy, this data storage and processing capacity was left for years to the individual institutions, with only a few Regions, such as Veneto (43) or Emilia-Romagna (44), that have built networks. However, this fragmented landscape should improve with the European Genomic Data Infrastructure initiative (45), that aims at creating an integrated infrastructure to support the storage, management, analysis, and sharing of genomic data across European Member States, although it requires continuous commitment and investments. Furthermore, this project should also help to establish common data standards and protocols to ensure that genomic data is consistent, high quality, and compatible across different systems that, together with the elaboration of national guidelines on the sequencing, analysis and clinical interpretation of data, missing to date, could promote interoperability, therefore enhancing research capabilities and the speed of scientific discovery (46).

This study has some strengths and limitations. To the best of our knowledge, this is the first self-assessment that was conducted in Italy regarding the use of genomics, allowing to draw an overview of the current stage of implementation in the most relevant domains. Indeed, baseline and ongoing measurements of key indicators are a cornerstone of implementing innovation (47), especially in complex systems such as healthcare (48). This study provides a state-of-the-art assessment of the implementation of genomic technology in Italy using an innovative tool. These results will be valuable in helping to define future Italian policies on genomics and, by extension, on innovative technologies such as artificial intelligence, with which they share common aspects. It has already served as an opportunity to discuss with relevant stakeholders both the state of the art and future steps, making this initiative a useful tool to support the elaboration of a national strategy. Furthermore, by testing the MLM in the Italian context, we laid the foundation for a monitoring procedure that tracks progress over time. The limitations of this study are mostly related to the MLM tool: despite the glossary provided, experts found some English wording to be ambiguous, and a few items were deemed to have partial applicability in the Italian NHS. However, by establishing common definitions during the consensus meetings, we were able to reach full agreement in all items, limiting this issue as much as possible. Secondly, none of the experts came from southern Italy, but since we included the NMG leaders that substantiated their claims with references to official documentation, and since the assessment was conducted from a national perspective, we believe our findings to be as accurate as possible. Lastly, we did not collect data at the regional level, as it was not the aim of our study, but this could be an interesting area for future research. In this regard, for future evaluations, we plan to: (i) increase the number of experts involved by recruiting participants from all over the country and (ii) reserve some spots for representatives of regional healthcare companies to enable data collection at sub-national level.

## Conclusion

Overall, the picture emerging from this survey is that of a country strongly convinced of both the crucial role that genomic applications have and will continue to have on the lives of citizens and patients, and the importance of implementing them according to the best practices of evidence-based medicine. However, although significant progress has been achieved in some areas at the national level, such as governance and the regulatory framework—particularly important in a decentralized system like the Italian NHS—an updated national strategy with coordinated actions and investment plans is needed to advance other key areas, especially healthcare worker education, public engagement, technical infrastructure, and clinical organization. In this regard, the strong commitment of at least part of the government could be instrumental in involving other relevant sectors, such as the Ministries of Economy, Education, and University, as well as other public and private stakeholders, to address the identified critical issues.

## Data Availability

The raw data supporting the conclusions of this article will be made available by the authors, without undue reservation.

## Footnotes

^1^ https://zenodo.org/records/8383706
